# Supplementary material for: LPS2336, a New TREK-1 Channel Activator Identified by High Throughput Screening
Source: Biomolecules. 2025 May 20;15(5):740. doi: 10.3390/biom15050740 (PMC12109561; doi:10.3390/biom15050740)
Supplement: Supplementary file 1 [file biomolecules-15-00740-s001.zip › biomolecules-3589624-supplementary.pdf]

Supplementary Materials:

LPS2336, a New TREK-1 Channel Activator Identified by High Throughput Screening

Romane Boyer [1], Romane Bony [1], Maxence Maugis [1], Julien Schopp [1], Marion Leroux [2], Clément Michelin [2], Laurie Gonthier [2], Quentin Grzeskiewicz [2], Alexandre Jouannet [2], Youssef Aissouni [1], Bruno Didier [3], Mihaela Gulea [3], Nicolas Girard [3], Jean-Christophe Cintrat [4], Antoine Dumeige [2], Jérôme Busserolles [1], Sylvie Ducki [2,*], Stéphane Lolignier [1,5,*]

[1] Université Clermont Auvergne, Inserm, CHU Clermont-Ferrand, Neuro-Dol, 63000 Clermont-Ferrand, France
[2] Université Clermont Auvergne, CNRS, Clermont Auvergne INP, ICCF, 63000 Clermont-Ferrand, France
[3] Université de Strasbourg, CNRS, Laboratoire d'Innovation Thérapeutique, LIT UMR 7200, 67000 Strasbourg, France
[4] Université Paris-Saclay, CEA, INRAE, Département Médicaments et Technologies pour la Santé (DMTS), SCBM, 91191 Gif-sur-Yvette, France
[5] Institut Universitaire de France (IUF), 75231 Paris, France
* Correspondence: stephane.lolignier@uca.fr; sylvie.ducki@sigma-clermont.fr

Supplementary Materials & Methods

Chemical sythesis

Compounds LPS4428, LPS4444, LPS4485, LPS4518, LPS4584, LPS4662, LPS4886, LPS6247, LPS6257, LPS7085, LPS7379, LPS7420 were kindly provided by Bruno Didier from the University of Strasbourg through a material transfer agreement (MTA) enabled by the French National Chemical Library, ChemBioFrance.

General

The chemical reagents and solvents were purchased from commercial sources and were used without further purification unless otherwise stated. Thin layer chromatography was performed on TLC glass backed silica Duracil UV_{254} (Macherey Nagel). Purification by Flash chromatography (FC) was performed with ARMEN Spot® and Chromabond RS 15 SiOH Macherey- Nagel. Purity was controlled by HPLC (Agilent® serie 1600 (254 and 280 nm)) and >95%. Conditions used for purity of the products; column: Proschell 120 EC-C18 2.7 µm (3.0 × 50 mm); temperature column: 40 °C; injection: 10 µL, 1 mL/min; solvents used A-water with 0.1% formic acid and B-Acetonitrile (95:5 to 5:95). Melting points were determined using a Büchi® apparatus and are given uncorrected. ^{1}H NMR and ^{13}C NMR spectra were recorded on a Bruker® Advanced spectrometer at 400 and 101 MHz. Chemical shifts δ are reported in ppm relative to unlabeled solvent. The coupling constants *J* are given in Hertz (Hz). The abbreviations used for signal descriptions are as s: singlet, brs: broad singlet, d: doublet, t: triplet, q: quartet, p: quintuplet. High Resolution Electro-Spray Ionisation Mass Spectra (HR-ESI-MS) were obtained from the from the "Service de Spectrométrie de masse du service UCA Partner, Université Clermont Auvergne" on UHPLC "Ultimate 3000" equipped with PDA detector, Q-exactive Orbitrap HRMS (thermoscientific). UHPLC purities are given as with the corresponding m/z peak, retention time (Rt) and %AUC of compound peak.

*Piperidine analogues **2a-k***

N-alkylation of piperidines **1a-c** (1 eq) with a halogenated compound (10 mmol) was carried out in the presence of potassium carbonate or DIPEA (1 eq) in acetone or DMF (0.5 M). The reaction mixture was heated under reflux for 2-5 hours, cooled down, filtered and purified by flash chromatography to afford the desired piperidine analogues **LPS2336** [1], **2b-h** and **2k** :

2-(4-Benzylpiperidin-1-yl)acetonitrile **LPS2336** was obtained from 4-benzylpiperidine **1a** (1.8 mL, 10.2 mmol), potassium carbonate (2.82 g), bromoacetonitrile (0.7 mL, 10.3 mmol) in acetone (20 mL) in 99% yield as a brown solid (2.1 g). CAS 25842-31-3, mp = 68-70 °C (lit 68 °C, *5a* in [2]) ; ^{1}H NMR (400 MHz, DMSO-d6) δ 7,27 (t, J = 8 Hz, 2H), 7,17 (t, J = 8 Hz, 3H), 3,66 (s, 2H), 2,74 (d, J = 11 Hz, 2H), 2,07 (td, J = 11, 3 Hz, 2H), 1,56 (d, J = 13 Hz, 2H), 1,51-1,43 (m, 1H), 1,19 (qd, J = 13, 3 Hz, 2H). ^{13}C NMR (101 MHz, DMSO-d6) δ 140,1 (C), 128,9 (CH), 128,1 (CH), 125,7 (CH), 115,8 (C), 51,6 (CH2), 45,4 (CH2), 42,1 (CH2), 36,4 (CH), 31,3 (CH2); LC-HRMS [ESI,m/z] 215.1542 (M+H, Rt 3.28 min, AUC 100%) calculated for $C_{14}H_{19}N_2$: 215.1543.
The salt **LPS2336.HCl** was prepared by stirring **LPS2336** (600 mg) in a solution of HCl in ethanol (10 eq, 1.25 M) at RT for 1 hour. The solid was filtered and washed with diethyl ether to obtain the salt as a white solid in 95% yield (669 mg) ; mp = 174-176 °C ; ^{1}H NMR (400 MHz, DMSO-d6) δ 7,29 (t, J = 8 Hz, 2H), 7,19 (t, J = 8 Hz, 3H), 4,49 (bs, 2H), 3.66 (m, 2H), 3,41 (d, J = 10 Hz, 2H), 2,97 (s, 2H), 1,77-1,74 (m, 3H), 1,59 (m, 2H) ; ^{13}C NMR (101 MHz, DMSO-d6) δ 139,4 (C), 128,9 (CH), 128,3 (CH), 126,0 (CH), 112,9 (C), 61,7 (CH2), 52.4 (CH2), 41,4 (CH2), 34,5 (CH), 28,4 (CH2).

3-(4-Benzylpiperidin-1-yl)propanenitrile **2a** was obtained from 4-benzylpiperidine **1a** (2.0 mL, 11.4 mmol), potassium carbonate (2.37 g, 17.1 mmol), bromopropionitrile (0.95 mL, 11.4 mmol) in acetone (23 mL) in 94% yield (2.4 g). CAS 4608-82-6 (*10k* in [2]) ^{1}H NMR (400 MHz, DMSO-d6) δ 7,31-7,27 (m, 2H), 7,21-7,16 (m, 3H), 3,44-3,35 (m, 2H), 3,20-3,16 (m, 2H), 2,95-2,82 (m, 2H), 1,73-1,67 (m, 2H), 1,61-1,55 (m, 2H), 1,19 (t, J = 7 Hz, 1H). ^{13}C NMR (101 MHz, DMSO-d6) δ 139,4 (C), 128,9 (CH), 128,2 (CH), 126,0 (CH), 117,9 (C), 60,5 (CH2), 51,5 (CH2), 41,4 (CH2), 35,0 (CH), 28,5 (CH2), 12,5 (CH2); LC-HRMS [ESI,m/z] 229.1695 (M+H, Rt 2.16 min, AUC 100%) calculated for $C_{15}H_{21}N_2$: 229.1699.

2-(4-Methylpiperidin-1-yl)acetonitrile **2b** was obtained from 4-methylpiperidine **1b** (1.03 g, 10.4 mmol), potassium carbonate (2.1 g), bromoacetonitrile (1.26 g, 10.4 mmol) in acetone (20 mL) in 91% yield (1.30 g). CAS 847574-01-0 (*6a* in [3]; ^{1}H NMR (400 MHz, DMSO-d6) δ 3.36 (s, 2H), 2.73 (d, J = 12 Hz, 2H), 2.11 (td, J = 12, 3 Hz, 2H), 1.61 (bd, J = 12 Hz, 2H), 1.31 (m, 1H), 1.13 (m, 2H), 0.89 (d, J = 7 Hz, 3H) ; ^{13}C NMR (101 MHz, DMSO-d6) 113.2 (C), 61.9 (CH2), 52.6 (CH2), 30.7 (CH2), 28.1 (CH), 21.2 (CH3) ; LC-HRMS [ESI,m/z] 139.1230 (M+H, Rt 0.71 min, AUC 99.5%) calculated for $C_8H_{15}N_2$: 139.1230.

4-Benzyl-1-(pent-2-yn-1-yl)piperidine **2c** was obtained from 4-benzylpiperidine **1a** (1.75 g, 10.0 mmol), potassium carbonate (2.1 g), 1-bromopent-2-yne (1.47 g, 10.0 mmol) in acetone (20 mL) in 53% yield (1.28 g). ^{1}H NMR (400 MHz, DMSO-d6) δ 7,29 (t, J = 8 Hz, 2H), 7,15 (m, 3H), 3,13 (bs, 2H), 2.72 (d, J = 12 Hz, 2H), 2,48 (m, 3H), 2.15 (m, 2H), 1,97 (dm, 2H), 1,52 (m, 2H), 1,41 (tm, 1H), 1.11-1.21 (m, 2H), 1.04 (t, J = 8 Hz, 3H) ; ^{13}C NMR (101 MHz, DMSO-d6) δ 139,5 (C), 129.0 (CH), 128,3 (CH), 126,0 (CH), 91,8 (C), 68,9 (C), 50,7 (CH2), 45,0 (CH2), 41,5 (CH2), 34,8 (CH), 28,5 (CH2), 13,1 (CH3), 11,7 (CH2) ; LC-HRMS [ESI,m/z] 242.1903 (M+H, Rt 2.84 min, AUC 100%) calculated for $C_{17}H_{24}N$: 242.1903.

2-(Piperidin-1-yl)acetonitrile **2d** was obtained from piperidine **1c** (0.83 g, 9.8 mmol), potassium carbonate (2.1 g), bromoacetonitrile (1.26 g, 10.4 mmol) in acetone (20 mL) in 99% yield (1.21 g). CAS

3010-03-5 (*9b* in [4]) ; ^{1}H NMR (400 MHz, DMSO-d6) δ 3.66 (s, 2H), 2.40 (m, 4H), 1.52 (m, 4H), 1.37 (3, 2H). ^{13}C NMR (101 MHz, DMSO-d6) δ 112.9 (C), 52,1 (CH2), 42,5 (CH2), 22,3 (CH2), 20.9 (CH2) ; LC-HRMS [ESI,m/z] 125.1075 (M+H, Rt 0.57 min, AUC 94%) calculated for $C_7H_{13}N_2$: 125.1072.

4-(4-Benzylpiperidin-1-yl)butanenitrile **2e** was obtained from 4-benzylpiperidine **1a** (2.0 g, 11.4 mmol), potassium carbonate (2.3 g), bromobutyronitrile (1.68 g, 11.4 mmol) in acetone (23 mL) in 95% yield (2.61 g). CAS 1098353-55-9 (*7a* in [2]) ; ^{1}H NMR (400 MHz, DMSO-d6) δ 7.27 (m, 2H), 7.16 (m, 3H), 2.77 (d, J = 12 Hz, 2H), 2.43-2.50 (m, 4H), 2.29 (t, J = 8 Hz, 2H), 1.79 (t, J = 8 Hz, 2H), 1.68 (m, 2H), 1.38-1.58 (m, 3H), 1.17 (m, 2H) ; ^{13}C NMR (101 MHz, DMSO-d6) δ 139.7 (C), 129.1 (CH), 128.4 (CH), 126.2 (CH), 119.9 (C), 54.6 (CH2), 51.8 (CH2), 41.7 (CH2), 35.2 (CH2), 28.7 (CH2), 19.6 (CH2), 14.3 (CH2) ; LC-HRMS [ESI,m/z] 243.1855 (M+H, Rt 2.22 min, AUC 100%) calculated for $C_{16}H_{23}N_2$: 243.1856.

1,4-Dibenzylpiperidine or N-benzyl-4-benzylpiperidine **2f** was obtained from 4-benzylpiperidine **1a** (1.8 g, 10.3 mmol), potassium carbonate (2.1 g), benzylbromide (1.2 mL g, 10.3 mmol) in acetone (20 mL) in 68% yield (1.96 g). CAS 193355-49-6 (*11* in [5]) ; ^{1}H NMR (400 MHz, DMSO-d6) δ 7.1-7.3 (m, 10H), 3.40 (s, 2H), 2.75 (d, J = 12 Hz, 2H), 2.48 (m, 2H), 1.83 (t J = 8 Hz, 2H), 1.42-1.57 (m, 3H), 1.11-1.26 (m, 2H) ; ^{13}C NMR (101 MHz, DMSO-d6) δ 139.6 (C), 131.7 (CH), 130.1 (C), 129.5 (CH), 129.1 (CH), 128.8 (CH), 128.5 (CH), 126.2 (CH), 59.1 (CH2), 51.4 (CH2), 41.7 (CH2), 35.3 (CH), 28.6 (CH2) ; LC-HRMS [ESI,m/z] 266.1903 (M+H, Rt 3.10 min, AUC 100%) calculated for $C_{19}H_{24}N$: 266.1903.

4-Benzyl-1-butylpiperidine **2g** was obtained from 4-benzylpiperidine **1a** (1.8 g, 10.3 mmol), potassium carbonate (2.1 g), 1-bromobutane (1.1 mL g, 10.3 mmol) in acetone (20 mL) in 60% yield (1.39 g). CAS 194798-06-6 (*4* in [6]) ; ^{1}H NMR (400 MHz, DMSO-d6) δ 7.25 (m, 2H), 7.15 (m, 3H), 2.76 (d, J = 12 Hz, 2H), 2.47 (m, 2H), 2.17 (t, J = 8 Hz, 2H), 1.72 (t, J = 8 Hz, 2H), 1.30-1.54 (m, 5H), 1.19-1.28 (m, 2H), 1.08-1.19 (m, 2H), 0.84 (t, J = 8 Hz, 3H) ^{13}C NMR (101 MHz, DMSO-d6) δ 140.0 (C), 129.4 (CH), 128.8 (CH), 126.5 (CH), 56.1 (CH2), 52.0 (CH2), 42.0 (CH2), 35.7 (CH), 29.1 (CH2), 25.5 (CH2), 20.0 (CH2), 14.0 (CH3) ; LC-HRMS [ESI,m/z] 232.2060 (M+H, Rt 2.73 min, AUC 91%) calculated for $C_{16}H_{26}N$: 232.2060.

Ethyl 2-(4-benzylpiperidin-1-yl)acetate **2h** was obtained from 4-benzylpiperidine **1a** (0.5 mL, 2.85 mmol), DIPEA (0.48 g), ethyl bromoacetate (0.32 mL g, 2.85 mmol) in DMF (5 mL) in 73% yield (544 mg). CAS 361979-66-0 (*1* in [7]) ; ^{1}H NMR (400 MHz, DMSO-d6) δ 7,26 (t, *J* = 8 Hz, 2H), 7,20 – 7,12 (m, 3H), 4,09 – 4,02 (m, 2H), 3,15 (s, 2H), 2,78 (d, *J* = 12 Hz, 2H), 2.51 (m, 2H), 2,12 – 1,97 (m, 2H), 1,50 (d, *J* = 12 Hz, 2H), 1,40 – 1,45 (m, 1H), 1,24 – 1,13 (m, 5H) ; ^{13}C NMR (101 MHz, DMSO-d6) δ : 170,2 (C), 140,4 (C), 129,1 (CH), 128,2 (CH), 125,8 (CH), 59,8 (CH2), 59,1 (CH2), 52,7 (CH2), 42,4 (CH2), 37.0 (CH), 31,7 (CH2), 14,2 (CH3) ; LC-HRMS [ESI,m/z] 262,1797 (M+H, Rt 2.63 min, AUC 100%) calculated for $C_{16}H_{24}NO_2$: 262,1802.

2-(4-Benzylpiperidin-1-yl)acetamide **2k** was obtained from 4-benzylpiperidine **1a** (100 mg, 0.57 mmol), potassium carbonate (113 mg, 1.14 mmol), a few mg of sodium iodide, bromoacetamide (94 mg, 0.69 mmol) in ethanol (2.5 mL) in 86% yield (114 mg). CAS 889604-07-3 (*1b* in [8]) ; ^{1}H NMR (400 MHz, DMSO-d6) δ 7,27 (t, *J* = 8 Hz, 2H), 7.18 (m, 3H), 7,09 (bs, 2H), 2.79 (m, 4H), 1,95 (t, *J* = 8 Hz, 2H), 1,51 (d, *J* = 12 Hz, 2H), 1,45 (m, 1H), 1,24 (d, *J* = 12 Hz, 2H). ^{13}C NMR (101 MHz, DMSO-d6) δ: 172.0 (C), 140,3 (C), 129.0 (CH), 128,1 (CH), 125,7 (CH), 61,8 (CH2), 53,6 (CH2), 42,4 (CH2), 36,9 (CH), 31,6 (CH2); LC-HRMS [ESI,m/z] 233.1646 (M+H, Rt 2.09 min, AUC 91.5%) calculated for $C_{14}H_{21}N_2O$: 233.1648.

2-(4-Benzylpiperidin-1-yl)acetic acid **2i** was obtained by saponification of ester **2h** (48 µL, 0.19 mmol) with potassium hydroxide (14 mg, 0.26 mmol) in methanol (1 mL) in 93% yield (42 mg). CAS 438634-

64-1 (*13c* in [1]) ; ^{1}H NMR (400 MHz, DMSO-d6) δ 7.26 (t, J = 12 Hz, 2H), 7,16 (m, 3H), 3,16 (s, 2H), 2,84 (d, *J* = 12 Hz, 2H), 2.67 (s, 1H), 2.33 (s, 1H), 1,78 (t, *J* = 12 Hz, 2H), 1,45-1,41 (m, 3H), 1,19 – 1,07 (m, 2H) ; ^{13}C NMR (101 MHz, DMSO-d6) δ 173.7 (C), 138.8 (C), 129.0 (CH), 128,1 (CH), 125,6 (CH), 65.0 (CH2), 63.1 (CH2), 53,3 (CH2), 37.5 (CH), 31,9 (CH2) ; LC-HRMS [ESI,m/z] 234.1482 (M+H, Rt 2.74 min, AUC 100%) calculated for $C_{14}H_{20}NO_2$: 234.1489.

Ethyl 2-(4-benzylpiperidin-1-yl)acetate **2j** was obtained by reduction of LPS2336 (100 mg, 0.47 mmol), with lithium aluminium hydride (42 mg, 1.12 mmol) in anhydrous THF (2 mL) at 0 °C for 24 hours, in 59% yield (60 mg). CAS 25842-32-4 (*15b* in [1]) ; ^{1}H NMR (400 MHz, $CDCl_3$) δ : 7.18-7.22 (m, 2H,), 7.06-7.13 (m, 3H), 2,80 (d, *J* = 12 Hz, 2H), 2,72 (t, *J* = 6 Hz, 2H), 2,46 (d, *J* = 6 Hz, 2H), 2,31 (t, *J* = 6 Hz, 2H), 1,84 (td, *J* = 12, 4 Hz, 2H), 1,64 (bs, 2H), 1,55 (d, *J* = 12 Hz, 2H), 1.45 (m, 1H), 1.19-1.28 (m, 2H) ; ^{13}C NMR (101 MHz, $CDCl_3$) δ : 140,9 (C), 129,2 (CH), 128,3 (CH), 125,9 (CH), 61,7 CH2), 54,2 (CH2), 43,4 (CH2), 39,3 (CH2), 38,1 (CH), 32,4 (CH2) ; LC-HRMS [ESI,m/z] 219.1854 (M+H, Rt 0.52 min, AUC 93%) calculated for $C_{14}H_{23}N_2$: 219.1856.

*Piperazine analogues **3a-d** and **4a-f***

1-Benzylpiperazine **3a** and 1,4-dibenzylpiperazine **3aa** were obtained from piperazine (1.007g, 11.7 mmol), benzyl bromide (230 µL, 2.34 mmol) in DCM (8 mL) stirred at 0 °C for 1 hour in respectively 33% (**3a,** 137 mg) and 1% (**3aa,** 27 mg) yield. The organic phase was washed with a saturated solution of sodium bicarbonate and dried over anhydrous magnesium sulphate. The products were separated by flash chromatography (SiO_2, DCM/MeOH/Et_3N) : **3a** (CAS 2759-28-6) : ^{1}H NMR (400 MHz, MeOH-d4) δ 7,23-7.35 (m, 5H), 3,52 (s, 2H), 2,84 (t, *J* = 8 Hz, 4H), 2,45 (bs, 4H) ; 13C NMR (101 MHz, MeOH-d4) δ 138,2 (C), 130,7 (CH), 129,3 (CH), 128,4 (CH), 64,4 (CH2), 54,4 (CH2), 46 (CH2) ; LC-HRMS [ESI,m/z] 177.1383 (M+H, Rt 0.51 min, AUC 91%) calculated for $C_{11}H_{17}N_2$: 177.1386. **3aa** (CAS 1034-11-3, *9* in [9]): mp 89-91 °C (lit 92 °C); ^{1}H NMR (400 MHz, MeOH-d4) δ 7,24-7.34 (m, 10H), 3,54 (s, 4H), 2,52 (bs, 8H); LC-HRMS [ESI,m/z] 267.1851 (M+H, Rt 2.15 min, AUC 100%) calculated for $C_{18}H_{23}N_2$: 267.1856.

To a solution of 2-, 3- or 4-(trifluoromethyl)benzyl bromide (1 eq) in toluene was added piperazine (4 eq). The mixture was stirred at 85 °C for 2 hours, cooled down to RT, filtered and purified by flash chromatography (SiO_2, DCM/EtOH/NH_4OH, 8/2/2, v/v/v) to afford the corresponding N-benzylated piperazines **3b**, **3c**, **3d** and **3dd**.

1-(4-trifluoromethyl)benzyl)piperazine **3b** was obtained from 1-(Bromomethyl)-4-(trifluoromethyl)benzene (300 mg, 1,26 mmol) in toluene (7.5 mL) and piperazine (432 mg, 5.02 mmol) as a white oil in 87% yield (267 mg). CAS 107890-32-4 (*33* in [10]); ^{1}H NMR (400 MHz, MeOH-d4) δ 7,62 (d, *J* = 8 Hz, 2H), 7,54 (d, *J* = 8 Hz, 2H), 3,59 (s, 2H), 2,84 (t, *J* = 8 Hz, 4H), 2,44 (bs, 4H) ; ^{13}C NMR (101 MHz, MeOH-d4) δ 143,5 (C), 131,0 (CH), 130,5 (C, d, J = 30 Hz), 126,1 (CH, q, J = 270 Hz), 126.2 (C, d, J= 4 Hz), 63,8 (CH2), 54,8 (CH2), 46,2 (CH2) ; LC-HRMS [ESI,m/z] 245.1255 (M+H, Rt 0.54 min, AUC 100%) calculated for $C_{12}H_{16}F_3N_2$: 245.1260.

1-(3-trifluoromethyl)benzyl)piperazine **3c** was obtained from 1-(Bromomethyl)-3-(trifluoromethyl)benzene (255 µL, 1,67 mmol) in toluene (10 mL) and piperazine (576 mg, 6.70 mmol) as a pinkish oil in 37% yield (150 mg). CAS 55513-16-1 (*32* in [10]) ; ^{1}H NMR (400 MHz, MeOH-d4) δ 7.51-7.67 (m, 4H), 3,60 (s, 2H), 2,86 (t, *J* = 8 Hz, 4H), 2,47 (bs, 4H) ; ^{13}C NMR (101 MHz, MeOH-d4) δ 140,2 (C), 134,2 (CH), 131,6 (C, q, J = 30 Hz), 130,1 (CH), 127.0 (CH, q, J = 270 Hz), 125,1 (C, q, J = 4 Hz), 63,7

(CH2), 54,4 (CH2), 46,1 (CH2) ; LC-HRMS [ESI,m/z] 245.1257 (M+H, Rt 1.54 min, AUC 99.3%) calculated for $C_{12}H_{16}F_3N_2$: 245.1260.

1-(2-trifluoromethyl)benzyl)piperazine **3d** was obtained from 1-(Bromomethyl)-2-(trifluoromethyl)benzene (260 µL, 1,67 mmol) in toluene (10 mL) and piperazine (576 mg, 6.70 mmol) as a pinkish oil in 74% yield (302 mg). CAS 94022-97-6 (*4* in [11]) ; ^1H NMR (400 MHz, MeOH-d4) δ 7,81 (d, *J* = 8 Hz, 1H), 7,65 (d, *J* = 8 Hz, 1H), 7,57 (t, *J* = 8 Hz, 1H), 7,39 (t, *J* = 8 Hz, 1H), 3,64 (s, 2H), 2,84 (m, 4H), 2,44 (s, 4H) ; ^{13}C NMR (101 MHz, MeOH-d4) δ 138,7 (C), 133,1 (CH), 131,9 (CH), 129,5 (C, q, J = 30 Hz), 128,3 (CH), 126,7 (CH, q, J = 4 Hz), 126.0 (C, q, J = 270 Hz), 59,9 (CH2), 54,9 (CH2), 46,4 (CH2) ; LC-HRMS [ESI,m/z] 245.1258 (M+H, Rt 2.11 min, AUC 100%) calculated for $C_{12}H_{16}F_3N_2$: 245.1260.

N-benzylated piperazines **3a-d** (1 eq) were subjected to N-alkylation by reaction with a halogenated compound (10 eq) in the presence of triethylamine (10 eq) in THF (1M). The mixture was stirred at RT for 2-5 hours, before quenching with a saturated sodium bicarbonate. Extraction with ethyl acetate afforded the crude product which was purified by flash chromatography (SiO_2, DMC/EtOAc) to afford the desired N,N′-disubstituted piperazines **4a-f**.

2-(4-benzylpiperazin-1-yl)acetonitrile **4a** was obtained from 1-benzylpiperazine (223 mg, 1.27 mmol) in THF (12 mL) in the presence of triethylamine (1.2 mL, 8.87 mmol) and chloroacetonitrile (0.8 mL, 12.68 mmol) in 43% yield (116 mg). CAS 92042-93-8 (*3* in [12]) ^1H NMR (400 MHz, MeOH-d4) δ 7.26-7.35 (m, 5H), 3,66 (s, 2H), 3,58 (s, 2H), 2.56-2.67 (m, 8H) ; ^{13}C NMR (101 MHz, MeOH-d4) δ : 137,9 (C), 130,7 (CH), 129,4 (CH), 128,6 (CH), 116,0 (C), 63,6 (CH2), 53,4 (CH2), 52,3 (CH2), 45,9 (CH2) ; LC-HRMS [ESI,m/z] 176.0026 (M-CH_2CN, Rt x,0.56 min, AUC 90%) calculated for $C_{11}H_{16}N_2$: 176.1313.

2-(4-(4-trifluoromethyl)benzyl)piperazin-1-yl)acetonitrile **4b** was obtained from 1-[[4-(trifluoromethyl)phenyl]methyl]-piperazine (239 mg, 0,98 mmol) in the presence of triethylamine (0.96 mL, 6,87 mmol) and chloroacetonitrile (0.62 mL, 9.81 mmol) in 55% yield (151 mg). CAS 1223410-40-9. mp 100-102 °C ; ^1H NMR (400 MHz, DMSO-d6) δ 7,69 (d, *J* = 8 Hz, 2H), 7,54 (d, *J* = 8 Hz, 2H), 3,72 (s, 2H), 3,57 (s, 2H), 2,42 (se, 8H) ; ^{13}C NMR (101 MHz, DMSO-d6) δ 143,3 (C), 129,3 (CH), 127,6 (C, d J = 30 Hz), 125,0 (CH, q, J = 4 Hz), 124,3 (C, q, J = 270 Hz), 115,8 (C), 61,1 (CH2), 52,1 (CH2), 51,1 (CH2), 45,0 (CH2) ; LC-HRMS [ESI,m/z] 284.1364 (M+H, Rt 2.14 min, AUC 100%) calculated for $C_{14}H_{17}F_3N_3$: 284.1369.

2-(4-(3-(trifluoromethyl)benzyl)piperazin-1-yl)acetonitrile **4c** was obtained from 1-[[3-(trifluoromethyl)phenyl]methyl]piperazine (168 µL, 0,82 mmol) in the presence of triethylamine (1.13 mL, 8.17 mmol) and chloroacetonitrile (0.77 mL, 12.51 mmol) in 49% yield (113 mg). CAS 1252352-52-5. ^1H NMR (400 MHz, MeOH-d4) δ 7,48 – 7,68 (m, 4H), 3,66 (s, 2H), 3,63 (s, 2H), 2,55-2,65 (m, 8H). ^{13}C NMR (101 MHz, MeOH-d4) δ 140, 3 (C), 134,2 (CH), 131,7 (C, q, J = 30 Hz), 130,2 (CH), 126,9 (CH, q, J = 4Hz), 125,7 (C, q, J = 270 Hz), 125,2 (CH, q, J= 4 Hz), 116,1 (C), 62,9 (CH2), 53,5 (CH2), 52,5 (CH2), 46.0 (CH2) ; LC-HRMS [ESI,m/z] 284.1363 (M+H, Rt 1.99 min, AUC 81%) calculated for $C_{14}H_{17}F_3N_3$: 284.1369. Traces of 1,4-bis(3-(trifluoromethyl)benzyl)piperazine **3cc** were identified by LC-HRMS [ESI,m/z] 403.1597 (M+H, Rt 3.79 min, AUC 19%) calculated for $C_{20}H_{21}F_6N_2$: 403.1603.

2-(4-(2-(trifluoromethyl)benzyl)piperazin-1-yl)acetonitrile **4d** was obtained from 1-[[2-(trifluoromethyl)phenyl]methyl]piperazine (196 mg, 0,80 mmol) in the presence of triethylamine (1.12 mL, 8.05 mmol) and chloroacetonitrile (0.51 mL, 8.05 mmol) in 93% yield (212 mg). mp 68-71 °C ; ^1H NMR (400 MHz, MeOH-d4) δ 7,84 (d, *J* = 8 Hz, 1H), 7,68 (d, *J* = 8 Hz, 1H), 7,61 (t, *J* = 8 Hz, 1H), 7,43 (t, *J*

= 8 Hz, 1H), 3,71 (s, 2H), 3,67 (s, 2H), 2,57-2.65 (m, 8H) ; ^{13}C NMR (101 MHz, MeOH-d4) δ 138,6 (C), 133,2 (CH), 131,8 (CH), 129,7 (C, d, J = 30 Hz), 128,3 (CH), 126,7 (CH, q, J = 4 Hz), 125,9 (C, q, J = 270 Hz), 116,1 (C), 59,1 (CH2), 53,7 (CH2), 52,8 (CH2), 46,0 (CH2) ; LC-HRMS [ESI,m/z] 284.1365 (M+H, Rt 1.97 min, AUC 96.3%) calculated for $C_{14}H_{17}F_3N_3$: 284.1374.

1-benzyl-4-(prop-2-yn-1-yl)piperazine **4e** was obtained from 1-benzylpiperazine (40 mg, 0.23 mmol) dissolved in DCM (1 mL) in the presence of triethylamine (63 µL, 0.45 mmol) and 3-bromoprop-1-yne (38 µL, 0.34 mmol) in 44% yield (22 mg). CAS 2521-00-8 (*k14* in [13]) ^{1}H NMR (400 MHz, MeOH-d4) δ 7.27-7.35 (m, 5H), 3,55 (s, 2H), 3,31 (s, 2H), 2,53-2.70 (m, 9H) ; ^{13}C NMR (101 MHz, MeOH-d4) δ 138,3 (C), 130.7 (CH), 129.3 (CH), 128.5 (CH), 79.0 (C), 75.2 (CH), 63.8 (CH2), 53,7 (CH2), 52,5 (CH2), 47.2 (CH2) ; LC-HRMS [ESI,m/z] 215.1539 (M+H, Rt 1.00 min, AUC 95.2%) calculated for $C_{14}H_{19}N_2$: 215.1543.

1-allyl-4-benzylpiperazine **4f** was obtained from 1-benzylpiperazine (100 mg, 0.39 mmol) dissolved in DCM (1 mL) in the presence of triethylamine (1.09 µL, 0.78 mmol) and 3-bromoprop-1-ene (37 µL, 0.43 mmol) in 61% yield (51 mg). CAS 336186-13-1 (*13* in [14]) ^{1}H NMR (400 MHz, $CDCl_3$) δ 7.15-7.25 (m, 5H), 3.78 (m, 1H), 5.09 (m, 2H), 3,45 (s, 2H), 2.93 (d, J = 8 Hz, 2H), 2.42 (bs, 8H) ; ^{13}C NMR (101 MHz, MeOH-d4) δ 138,3 (C), 135.2 (CH), 130.6 (CH), 129.3 (CH), 128.4 (CH), 119.4 (CH2), 63.8 (CH2), 62.5 (CH2), 53.6 (CH2), 53.5 (CH2) ; LC-HRMS [ESI,m/z] 217.1692 (M+H, Rt 0.89 min, AUC 100%) calculated for $C_{14}H_{21}N_2$: 217.1699.

References

1. Contreras, J.-M.; Parrot, I.; Sippl, W.; Rival, Y.M.; Wermuth, C.G. Design, Synthesis, and Structure−Activity Relationships of a Series of 3-[2-(1-Benzylpiperidin-4-Yl)Ethylamino]Pyridazine Derivatives as Acetylcholinesterase Inhibitors. *J. Med. Chem.* **2001**, *44*, 2707–2718. https://doi.org/10.1021/jm001088u.
2. Amata, E.; Dichiara, M.; Arena, E.; Pittalà, V.; Pistarà, V.; Cardile, V.; Graziano, A.C.E.; Fraix, A.; Marrazzo, A.; Sortino, S.; et al. Novel Sigma Receptor Ligand–Nitric Oxide Photodonors: Molecular Hybrids for Double-Targeted Antiproliferative Effect. *J. Med. Chem.* **2017**, *60*, 9531–9544. https://doi.org/10.1021/acs.jmedchem.7b00791.
3. Ruchelman, A.L.; Houghton, P.J.; Zhou, N.; Liu, A.; Liu, L.F.; LaVoie, E.J. 5-(2-Aminoethyl)Dibenzo[c,h][1,6]Naphthyridin-6-Ones: Variation of N-Alkyl Substituents Modulates Sensitivity to Efflux Transporters Associated with Multidrug Resistance. *J. Med. Chem.* **2005**, *48*, 792–804. https://doi.org/10.1021/jm049447z.
4. Chiba, T.; Takata, Y. Anodic Cyanation of Tertiary Aliphatic and Heterocyclic Amines. *J. Org. Chem.* **1977**, *42*, 2973–2977. https://doi.org/10.1021/jo00438a005.
5. McGonagle, F.I.; MacMillan, D.S.; Murray, J.; Sneddon, H.F.; Jamieson, C.; Watson, A.J.B. Development of a Solvent Selection Guide for Aldehyde-Based Direct Reductive Amination Processes. *Green Chem.* **2013**, *15*, 1159–1165. https://doi.org/10.1039/C3GC40359A.
6. Cai, J.; Wathey, B. A Novel Traceless Solid Phase Tertiary Amine Synthesis Based on Merrifield *Resin. Tetrahedron Letters* **2001**, *42*, 1383–1385. https://doi.org/10.1016/S0040-4039(00)02251-6.
7. Özturan Özer, E.; Tan, O.U.; Ozadali, K.; Küçükkılınç, T.; Balkan, A.; Uçar, G. Synthesis, Molecular Modeling and Evaluation of Novel N′-2-(4-Benzylpiperidin-/Piperazin-1-Yl)Acylhydrazone Derivatives as Dual Inhibitors for Cholinesterases and Aβ Aggregation. *Bioorg. Med. Chem. Lett.* **2013**, *23*, 440–443. https://doi.org/10.1016/j.bmcl.2012.11.064.
8. Chaudhari, K.H.; Mahajan, U.S.; Bhalerao, D.S.; Akamanchi, K.G. Novel and Facile Transformation of N,N-Disubstituted Glycylamides into Corresponding Cyanamides by Using Pentavalent Iodine Reagents in Combination with Tetraethylammonium Bromide. *Synlett* **2007**, *18*, 2815–2818. https://doi.org/10.1055/s-2007-991093.

9. Putt, K.S.; Chen, G.W.; Pearson, J.M.; Sandhorst, J.S.; Hoagland, M.S.; Kwon, J.-T.; Hwang, S.-K.; Jin, H.; Churchwell, M.I.; Cho, M.-H.; et al. Small-Molecule Activation of Procaspase-3 to Caspase-3 as a Personalized Anticancer Strategy. *Nat. Chem. Biol.* **2006**, *2*, 543–550. https://doi.org/10.1038/nchembio814.
10. Ferla, S.; Manganaro, R.; Benato, S.; Paulissen, J.; Neyts, J.; Jochmans, D.; Brancale, A.; Bassetto, M. Rational Modifications, Synthesis and Biological Evaluation of New Potential Antivirals for RSV Designed to Target the M2-1 Protein. *Bioorg. Med. Chem.* **2020**, *28*, 115401. https://doi.org/10.1016/j.bmc.2020.115401.
11. Liu, G.; Lynch, J.K.; Freeman, J.; Liu, B.; Xin, Z.; Zhao, H.; Serby, M.D.; Kym, P.R.; Suhar, T.S.; Smith, H.T.; et al. Discovery of Potent, Selective, Orally Bioavailable Stearoyl-CoA Desaturase 1 Inhibitors. *J. Med. Chem.* **2007**, *50*, 3086–3100. https://doi.org/10.1021/jm070219p.
12. Saxena, M.; Agarwal, S.K.; Patnaik, G.K.; Saxena, A.K. Synthesis, Biological Evaluation, and Quantitative Structure-Activity Relationship Analysis of [.Beta.-(Aroylamino)Ethyl]Piperazines and -Piperidines and [2-[(Arylamino)Carbonyl]Ethyl]Piperazines, -Piperidines, -Pyrazinopyridoindoles, and -Pyrazinoisoquinolines. A New Class of Potent H1 Antagonists. *J. Med. Chem.* **1990**, *33*, 2970–2976. https://doi.org/10.1021/jm00173a011.
13. Jia, Z.; Wen, H.; Huang, S.; Luo, Y.; Gao, J.; Wang, R.; Wan, K.; Xue, W. "Click" Assembly of Novel Dual Inhibitors of AChE and MAO-B from Pyridoxine Derivatives for the Treatment of Alzheimer's Disease. *Heterocycl. Commun.* **2022**, *28*, 18–25. https://doi.org/10.1515/hc-2022-0002.
14. Murty, M.S.R.; Jyothirmai, B.; Radha Krishna, P.; Yadav, J.S. Zinc Mediated Alkylation of Cyclic Secondary Amines. *Synth. Commun.* **2003**, *33*, 2483–2486. https://doi.org/10.1081/SCC-120021838.
